# A Genome-Wide Survey on Basic Helix-Loop-Helix Transcription Factors in Giant Panda

**DOI:** 10.1371/journal.pone.0026878

**Published:** 2011-11-09

**Authors:** Chunwang Dang, Yong Wang, Debao Zhang, Qin Yao, Keping Chen

**Affiliations:** 1 Institute of Life Sciences, Jiangsu University, Zhenjiang, Jiangsu Province, People's Republic of China; 2 School of Food and Biological Engineering, Jiangsu University, Zhenjiang, Jiangsu Province, People's Republic of China; University of South Florida College of Medicine, United States of America

## Abstract

The giant panda (*Ailuropoda melanoleuca*) is a critically endangered mammalian species. Studies on functions of regulatory proteins involved in developmental processes would facilitate understanding of specific behavior in giant panda. The basic helix-loop-helix (bHLH) proteins play essential roles in a wide range of developmental processes in higher organisms. bHLH family members have been identified in over 20 organisms, including fruit fly, zebrafish, mouse and human. Our present study identified 107 bHLH family members being encoded in giant panda genome. Phylogenetic analyses revealed that they belong to 44 bHLH families with 46, 25, 15, 4, 11 and 3 members in group A, B, C, D, E and F, respectively, while the remaining 3 members were assigned into “orphan”. Compared to mouse, the giant panda does not encode seven bHLH proteins namely Beta3a, Mesp2, Sclerax, S-Myc, Hes5 (or Hes6), EBF4 and Orphan 1. These results provide useful background information for future studies on structure and function of bHLH proteins in the regulation of giant panda development.

## Introduction

The basic helix-loop-helix (bHLH) proteins form a large superfamily of transcription factors that play crucial roles in a wide range of developmental processes including neurogenesis, myogenesis, hematopoiesis, sex determination and gut development. The bHLH domain is approximately 60 amino acids long and comprises a DNA-binding basic region (b) and two helices separated by a variable loop region (HLH) [1]. The HLH domain promotes dimerization, allowing the formation of homodimeric or heterodimeric complexes between different bHLH family members. The two basic domains which are brought together through dimerization bind specific hexanucleotide sequences.

In the past two decades, protein functions of animal bHLH family members have been well characterized mainly through studies on bHLH proteins in model organisms including the nematode (*Caenorhabditis elegans*), fruit fly (*Drosophila melanogaster*) and mouse (*Mus musculus*). It has been established that animal bHLHs are classified into 45 families based on their different functions in the regulation of gene expression. In addition, they are divided into 6 groups according to target DNA elements they bind and their own structural characteristics. Specifically, group A consists of 22 families. They mainly regulate neurogenesis, myogenesis and mesoderm formation. Group B consists of 12 families. They mainly regulate cell proliferation and differentiation, sterol metabolism and adipocyte formation, and expression of glucose-responsive genes. Group C has 7 families. They are responsible for the regulation of midline and tracheal development, circadian rhythms, and for the activation of gene transcription in response to environmental toxins. Group D has only 1 family. It forms inactive heterodimers with group A bHLH proteins. Group E has 2 families, which regulate embryonic segmentation, somitogenesis and organogenesis etc. Group F also has 1 family. It regulates head development and formation of olfactory sensory neurons etc (reviewed in [2]).

With the completion of genome sequencing projects for an increased number of organisms, bHLH family members have been identified in genomes of over 20 organisms. These include 8 bHLH genes in *Saccharomyces cerevisiae*, 16 in *Amphimedon queenslandica*, 33 in *Hydra magnipapillata*, 42 in *Caenorhabditis elegans*, 46 in *Ciona intestinalis*, 50 in *Strongylocentrotus purpuratus*, 51 in *Apis mellifera*, 52 in *Bombyx mori*, 57 in *Daphia pulex*, 59 in *Drosophila melanogaster*, 63 in *Lottia gigantea*, 64 in *Capitella* sp 1, 68 in *Nematodtella vectensis*, 78 in *Branchiostoma floridae*, 87 in *Tetraodon nigroviridis*, 104 in *Gallus gallus*, 114 in *Mus musculus*, 114 in *Rattus norvegicus*, 118 in *Homo sapiens*, 139 in *Brachydanio rerio*, 147 in *Arabidopsis*, and 167 in *Oryza sativa* [3]–[12].

The giant panda, *Ailuropoda melanoleuca*, is a critically endangered mammal confined in six isolated mountain ranges of South-western China [13]. As one of the most primitive carnivores, giant panda not only has unique food habit, but also has highly specialized reproductive behavior and low fertility [14], all of which signify that the giant panda has considerably different regulatory mechanisms in growth and development. However, very little is known on structure and function of regulatory genes in the growth and development of giant panda [15], [16]. As bHLH proteins present great importance in the regulation of organismal development, in this study, we have made exhaustive effort to obtain the complete list of bHLH family members encoded in the genome of giant panda. As a result, 107 bHLH family members were identified. Phylogenetic analyses with their mouse bHLH homologues revealed that the 107 giant panda bHLH members belong to 44 bHLH families with 46, 25, 15, 4, 11 and 3 members in group A, B, C, D, E and F, respectively, while 3 members were assigned into “orphan”. The present study provides useful background information for future studies on structure and function of bHLH proteins in the regulation of giant panda development.

## Materials and Methods

### Blast Searches

The sets of 45 representative bHLH domains and 114 mouse bHLH motifs were from the additional files of previous reports [4], [17], respectively. Each sequence of both sets was used as query sequence to perform tblastn search against the giant panda genome sequences which were accessed through the hyperlink provided on GenBank's MapView webpage (http://www.ncbi.nlm.nih.gov/mapview/). The expect value (*E*) was set at 10 in order to obtain all bHLH related sequences. The obtained subject sequences were manually examined to keep only one sequence for those that have the same contig number, reading frame and coding regions, to add the missing amino acids to corresponding sites with the help of EditSeq program (version 5.01) of the DNAStar package, and to find introns within the bHLH motifs using NetGene2 application online (http://www.cbs.dtu.dk/services/NetGene2/). Sequence accession numbers of giant panda bHLH proteins were obtained by using amino acids of each identified bHLH motif to conduct blastp search against giant panda protein sequence databases which were also accessed through the hyperlink on GenBank's MapView webpage.

### Sequence Alignment

All sequences that had been improved by the above methods were aligned using ClustalW program embedded in MEGA4 [18] with default settings. Each sequence was examined for their amino acid residues at the 19 conserved sites by manual checking [19]. Sequences with less than nine variations were regarded as potential giant panda bHLH members. The sequences which have less than ten conserved amino acids were discarded and the rest sequences were aligned again using ClustalW. The aligned giant panda bHLH motifs were shaded in GeneDoc Multiple Sequence Alignment Editor and Shading Utility (Version 2.6.02) [20] and copied to rich text file for further annotation.

### Phylogenetic Analyses

Phylogenetic analyses to all the identified giant panda bHLH members were carried out in two steps. First, all the obtained giant panda bHLH motif sequences were used to build neighbor-joining (NJ) distance tree with the 114 mouse bHLH motif sequences using PAUP 4.0 Beta 10 [21] based on a step matrix constructed from Dayhoff PAM 250 distance matrix by R. K. Kuzoff (http://paup.csit.fsu.edu/nfiles.html). Then, each giant panda bHLH motif sequence was used to conduct in-group phylogenetic analyses [9] with mouse bHLH motif sequences. That is, each amino acid sequence of giant panda bHLH motifs was used to construct NJ, maximum parsimony (MP), and maximum likelihood (ML) phylogenetic trees with mouse bHLH family members of the corresponding group, respectively. The NJ trees were bootstrapped with 1,000 replicates to provide information about their statistical reliability. MP analysis was performed using heuristic searches and bootstrapped with 100 replicates. ML trees were constructed using TreePuzzle 5.2 [22] with quartet-puzzling tree-search procedure and 25,000 puzzling steps. Model of substitution was set to the Jones-Taylor-Thornton [23]. Other parameters were set to default values.

## Results and Discussion

### Giant Panda bHLH Family Members

The tblastn searches, sequence alignment, and examination of the 19 conserved amino acid sites revealed that there were 107 *bHLH* genes encoded in giant panda genome. The names of all 107 giant panda bHLH members are listed in Table 1. Each identified giant panda *bHLH* (*GpbHLH*) gene was named according to nomenclature used by mouse bHLH sequences. The alignment of all 107 GpbHLH motifs is shown in Figure S1 and the phylogenetic tree constructed using amino acids from 107 GpbHLH motifs and 114 mouse bHLH motifs is shown in Figure S2. Figures S1 and S2 together show that there were 46, 25, 15, 4, 11 and 3 members in group A, B, C, D, E and F, respectively. And additional 3 members were assigned into “orphan”. We found that gene encoding for member of Delilah family was not found in the giant panda genome. In Figure S1, there are two most conserved sites located at sites 23 and 59 of the bHLH motif. Besides, there are other eight sites which are also conserved as indicated with asterisks on top of Figure S1 (amino acid sequences of all 107 giant panda bHLH motifs are available in file S1).

**Table 1:** A complete list of bHLH genes from giant panda.

Family	Gene name	Mouse homologue	Bootstrap values			Protein accession number	Annotation in GenBank
			NJ	MP	ML		
ASCa	*GpAsh1*	*Mash1*	99	92	99	XP_002915515.1	Hypothetical protein
	*GpAsh2*	*Mash2*	92	91	97	XP_002920180.1	Ash2
ASCb	*GpAsh3a*	*Mash3a*	98	99	90	XP_002916197.1	Ash3a
	*GpAsh3b*	*Mash3b*	98	97	100	hmm367624.p	Hypothetical protein
	*GpAsh3c*	*Mash3c*	99	97	71	hmm285394.p	Hypothetical protein
MyoD	*GpMyoD*	*MyoD*	99	97	94	XP_002928807.1	MyoD
	*GpMyoG*	*MyoG*	100	98	96	XP_002925479.1	MyoG
	*GpMyf5*	*Myf5*	99	77	78	XP_002916822.1	Myf5
	*GpMyf6*	*Myf6*	99	89	78	XP_002916823.1	Myf6
E12/E47	*GpTF12*	*TF12*	82	n/m*	n/m*	XP_002920720.1	TF12
	*GpE2A*	*E2A*	100	97	98	XP_002923565.1	E2A
	*GpKA1*	*KA1*	65	87	57	Not available	/
	*GpTCF4*	*TCF4*	90	21	82	XP_002914713.1	TCF4
Ngn	*GpAth4a*	*Math4a*	99	95	97	XP_002926036.1	Neurogenin-2-like
	*GpAth4b*	*Math4b*	99	93	99	XP_002913770.1	Neurogenin-3-like
	*GpAth4c*	*Math4c*	99	85	99	XP_002913012.1	Neurogenin-1-like
NeuroD	*GpNDF1*	*NDF1*	89	27	80	XP_002922319.1	NDF1
	*GpNDF2*	*NDF2*	88	68	89	XP_002916875.1	NDF2
	*GpAth2*	*Math2*	93	77	89	XP_002919308.1	NDF6
	*GpAth3*	*Math3*	99	96	98	XP_002930692.1	NDF4
Atonal	*GpAth1*	*Math1*	100	99	100	XP_002915330.1	Ath1
	*GpAth5*	*Math5*	100	100	100	XP_002913786.1	Ath7
Mist	*GpMist1*	*Mist1*	99	97	n/m	Not available	/
Beta3	*GpBeta3b* a	*Beta3b*	100	59	n/m*	XP_002925784.1	Class E bHLH protein 23
Oligo	*GpOligo1*	*Oligo1*	91	85	98	XP_002919636.1	Oligo1
	*GpOligo2*	*Oligo2*	88	59	56	XP_002919637.1	Oligo2
	*GpOligo3*	*Oligo3*	90	73	98	XP_002915132.1	Oligo3
Net	*GpAth6*	*Math6*	100	100	100	XP_002928677.1	Ath8
Mesp	*GpMesp1* a	*Mesp1*	99	78	n/m*	XP_002919616.1	Mesp1
	*GpPMeso1*	*pMeso1*	100	100	97	XP_002915045.1	Mesogenin-1
Twist	*GpTwist*	*Twist*	91	64	n/m*	XP_002915415.1	Hypothetical protein
	*GpDermo1*	*Dermo1*	90	55	90	XP_002922521.1	Twist-2-like
Paraxis	*GpParaxis*	*Paraxis*	78	70	86	XP_002925450.1	Transcription factor 15
MyoRa	*GpMyoR*	*MyoR*	75	64	84	XP_002922844.1	Musculin
	*GpPod1*	*Pod1*	78	25	n/m*	XP_002922333.1	Transcription factor 21
MyoRb	*GpMyoRb1*	*MyoRb1*	100	100	100	XP_002916432.1	Hypothetical protein
	*GpMyoRb2*	*MyoRb2*	100	99	93	XP_002913861.1	Transcription factor 23
Hand	*GpDHand*	*dHand*	100	64	n/m*	XP_002912726.1	Hand 2
	*GpEHand*	*eHand*	99	99	99	XP_002917201.1	Hand 1
PTFa	*GpPTFa*	*PTFa*	100	100	97	XP_002913208.1	Hypothetical protein
PTFb	*GpPTFb*	*PTFb*	100	100	99	XP_002915418.1	Fer3
SCL	*GpTal1*	*Tal1*	100	71	85	hmm534354.p	Hypothetical protein
	*GpTal2*	*Tal2*	99	94	88	XP_002927719.1	Tal2
	*GpLyl1*	*Lyl1*	99	99	100	XP_002921032.1	Lyl1
NSCL	*GpHen1* a	*Hen1*	100	100	89	XP_002928490.1	HLH protein-1-like
	*GpHen2*	*Hen2*	60	40	n/m*	XP_002925359.1	HLH protein-2-like
SRC	*GpSRC1*	*SRC1*	100	99	98	XP_002913840.1	NcoA 1
	*GpSRC2*	*SRC2*	100	100	99	XP_002918551.1	NcoA 2
	*GpSRC3*	*SRC3*	100	100	99	XP_002927046.1	NcoA 3
FIGα	*GpFiga*	*Figa*	100	100	100	XP_002914962.1	Figa
Myc	*GpN-Myc*	*N-Myc*	99	70	85	XP_002923116.1	N-Myc
	*GpC-Myc*	*C-Myc*	100	100	100	XP_002915028.1	C-Myc
	*GpL-Myc*	*L-Myc*	100	100	99	XP_002927604.1	L-Myc
Mad	*GpMxi1*	*Mxi1*	100	99	96	XP_002930845.1	Mxi1
	*GpMad1*	*Mad1*	100	100	92	XP_002914951.1	Mad1
	*GpMad3*	*Mad3*	100	98	91	XP_002928184.1	Mad3
	*GpMad4*	*Mad4*	98	87	91	XP_002916603.1	Mad4
Mnt	*GpMnt*	*Mnt*	100	100	99	XP_002918069.1	Mnt
Max	*GpMax*	*Max*	100	100	100	XP_002914193.1	Max
USF	*GpUSF1*	*USF1*	100	98	100	XP_002928795.1	USF1
	*GpUSF2*	*USF2*	92	38	99	XP_002920933.1	USF2
MITF	*GpMITF*	*MITF*	75	n/m*	n/m*	XP_002927657.1	MITF
	*GpTFEb*	*TFEb*	100	100	98	XP_002914561.1	TFEb
	*GpTFEc*	*TFEc*	98	97	96	XP_002923929.1	TFEc
	*GpTFE3*	*TFE3*	63	52	n/m	XP_002917800.1	TFE3
SREBP	*GpSREBP1*	*SREBP1*	100	99	n/m*	XP_002923179.1	SREBP1
	*GpSREBP2*	*SREBP2*	100	100	100	XP_002929331.1	SREBP2
AP4	*GpAP4*	*AP4*	100	100	99	XP_002924645.1	AP4
MLX	*GpMlx*	*Mlx*	100	99	92	XP_002923532.1	WBSCR14
	*GpMondoA*	*MondoA*	100	100	97	XP_002913172.1	Mlx-interacting protein
TF4	*GpTF4*	*TF4*	100	100	100	XP_002922185.1	Max-like protein X
Clock	*GpClk*	*Clk*	100	95	100	XP_002919413.1	Clk
	*GpNPAS2*	*NPAS2*	100	99	100	XP_002919235.1	NPAS2
ARNT	*GpARNT1*	*ARNT1*	97	61	n/m*	XP_002919403.1	ARNT1
	*GpARNT2*	*ARNT2*	96	87	97	XP_002919129.1	ARNT2
Bmal	*GpBmal1*	*Bmal1*	100	99	97	XP_002926157.1	ARNT-like protein 1
	*GpBmal2* a	*Bmal2*	100	100	100	XP_002917162.1	ARNT-like protein 2
Sim	*GpSim1*	*Sim1*	97	74	96	XP_002922016.1	Sim1
	*GpSim2*	*Sim2*	97	83	90	hmm348774.p	Hypothetical protein
AHR	*GpAHR1*	*AHR1*	100	99	100	XP_002917450.1	AHR1
	*GpAHR2*	*AHR2*	81	64	n/m	XP_002926684.1	AHRR
Trh	*GpNPAS3*	*NPAS3*	100	91	82	hmm740504.p	Hypothetical protein
HIF	*GpHif1a*	*Hif1a*	100	100	100	XP_002913080.1	Hif1a
	*GpHif3a*	*Hif3a*	100	100	100	XP_002923099.1	Hif3a
	*GpNPAS1*	*NPAS1*	100	100	95	XP_002923107.1	NPAS1
	*GpEPAS1*	*EPAS1*	100	99	100	XP_002912483.1	EPAS1
Emc	*GpId1*	*Id1*	93	57	n/m*	hmm387023.p	Hypothetical protein
	*GpId2*	*Id2*	87	82	56	XP_002923275.1	Id2
	*GpId3*	*Id3*	99	92	100	XP_002913316.1	Id3
	*GpId4*	*Id4*	100	90	76	hmm7844.p	Hypothetical protein
Hey	*GpHerp1*	*Herp1*	96	86	96	XP_002927896.1	Herp1
	*GpHerp2*	*Herp2*	96	50	n/m*	XP_002915182.1	Herp2
	*GpHEYL*	*HEYL*	98	94	98	XP_002930399.1	HEYL
	*GpHey4*	*Hey4*	100	100	92	XP_002914075.1	HELT-like protein
H/E(spl)	*GpDec1*	*Dec1*	76	67	99	XP_002920034.1	Class E bHLH protein 40
	*GpDec2*	*Dec2*	73	n/m*	n/m*	hmm164814.p	Hypothetical protein
	*GpHes1a*	*Hes1*	99	66	84	XP_002930213.1	Hes4-like protein
	*GpHes1b*	*Hes1*	100	100	100	XP_002923794.1	Hes1
	*GpHes2*	*Hes2*	97	67	88	XP_002923913.1	Hes2
	*GpHes3*	*Hes3*	100	97	98	XP_002923915.1	Hes3
	*GpHes7*	*Hes7*	100	100	97	hmm475304.p	Hypothetical protein
COE	*GpEBF1*	*EBF1*	95	45	58	XP_002912553.1	COE1
	*GpEBF2*	*EBF2*	94	89	56	XP_002914472.1	COE2
	*GpEBF3*	*EBF3*	72	n/m*	n/m*	XP_002922830.1	COE3
Orphan	*GpOrphan2*	*Orphan2*	100	100	59	XP_002913251.1	MAX gene-associated
	*GpOrphan3*	*Orphan3*	100	100	n/m*	XP_002923506.1	Sohlh2-like protein
	*GpOrphan4*	*Orphan4*	100	100	100	Not available	/

**Note:** Giant panda bHLH genes were named according to their mouse homologues. Bootstrap values were from in-group phylogenetic analyses with mouse bHLH motif sequences using NJ, MP, and ML algorithms, respectively. OsRa (the rice bHLH motif sequence of R family) was used as the outgroup in every constructed tree except those for *GpHen1* and *GpBmal2* which used separate outgroup sequence. n/m means that a giant panda bHLH does not form a monophyletic group with any other single bHLH motif sequence. n/m* means that a giant panda bHLH does not form a monophyletic clade with any specific bHLH motif sequence but forms a monophyletic clade with other bHLH proteins of the same family.

a means that the gene's orthology was defined by in-group phylogenetic analyses with corresponding whole bHLH protein sequences from mouse. The accession numbers are from different protein resources. Those labeled as “XP” are from ‘RefSeq protein’ and those labeled as “hmm” were from ‘*Ab initio* protein’ databases of giant panda.

### Identification of Orthologous Families

Ortholog identification has had much uncertainty since there is no absolute criterion that can be used to decide whether two genes are orthologous [17]. In our previous studies [9], [10], in-group phylogenetic analysis was adopted to identify homologues for the unknown sequences that would form a monophyletic clade among themselves by using a more certain criterion based on the criterion used by Ledent et al. [17], [24]: If an unknown single giant panda bHLH forms a monophyletic clade with another bHLH of known family in phylogenetic trees constructed with different methods and all the bootstrap values exceed 50, the known member will be regarded as a homologue of the unknown sequence. Figure S3, as an example here, shows NJ, MP and ML phylogenetic trees constructed with one GpbHLH member (GpAsh1) and eight group A bHLH members from mouse. In all three trees, GpAsh1 formed monophyletic clade with Mash1 of mouse with bootstrap values ranging from 92 to 100. Therefore, GpAsh1 was considered as an ortholog of Mash1 of mouse. The similar in-group phylogenetic analyses were conducted to each of the identified GpbHLH members by referencing Figure S2 to select appropriate related mouse bHLH members for the analysis. All the bootstrap values of constructed NJ, MP and ML trees were listed in Table 1 without showing the correspondent constructed trees. Table 1 showed that the orthology of GpbHLH members with mouse can be divided into the following categories.

Firstly, among the 107 GpbHLH members, 83 GpbHLH members have all the bootstrap values over 50 (55≦bootstrap values≦100) in constructed NJ, MP and ML trees. We have sufficient confidence to define orthology of these GpbHLH motifs to their corresponding mouse bHLH orthologs.

Secondly, 4 GpbHLH members, namely *GpTCF4*, *GpNDF1*, *GpUSF2* and *GpEBF1*, formed monophyletic clade with bootstrap values over 50 in NJ and ML trees. Although they also formed monophyletic clade in MP trees, their bootstrap values ranged from 21 to 45. Therefore, the orthology of these 4 GpbHLH members have been defined according to the statistical support from NJ and ML trees. And 10 GpbHLH members, namely *GpMist1*, *GpAHR2*, *GpTwist*, *GpDHand*, *GpARNT1*, *GpSREBP1*, *GpId1*, *GpHerp2* and *GpOrphan3*, formed monophyletic clade with bootstrap values ranging from 50 to 100 in NJ and MP trees, but did not form monophyletic group with any single bHLH sequence in ML trees (marked with n/m* or n/m in Table 1). For these 9 GpbHLH members, we have defined their orthology according to the statistical support from NJ and MP trees.

Thirdly, 2 GpbHLH members, namely *GpPod1* and *GpHen2* formed monophyletic clade in NJ and MP trees with bootstrap values ranging from 20 to 79 but did not form monophyletic group in ML tree. And 4 other GpbHLH members, namely *GpTF12*, *GpMITF*, *GpDec2* and *GpEBF3*, formed monophyletic clade with bootstrap values ranging from 72 to 82 in NJ tree, but did not form monophyletic clade in MP and ML trees. Although these 6 GpbHLH members did not have sufficient bootstrap support, we defined orthologs for them because they all have one or two bootstrap support to testify their orthology to the correspondent mouse ortholog. This phylogenetic divergence of bHLH motif sequences between giant panda and mouse probably means that these two mammals have evolved in quite different circumstances.

Finally, there are 4 GpbHLH sequences which did not form monophyletic clade with most of the mouse bHLH motif sequences in all constructed phylogenetic trees. They are *GpBeta3b*, *GpMesp1*, *GpHen1* and *GpBmal2* of which whole protein sequences were used to conduct in-group phylogenetic analyses with whole sequences of corresponding mouse bHLH proteins for defining their orthology (marked with ^a^ in Table 1).

### Protein Sequences and Genomic Coding Regions of Giant Panda *bHLH* Genes

Protein sequence accession numbers of all the identified GpbHLH motifs were listed in Table 1. It was found that there are 95 GpbHLH motifs of which protein sequence accession numbers were found in ‘Non-RefSeq protein’ database (shown as ‘XP’ plus number). Protein sequence accession numbers of 9 GpbHLH motifs were only found in ‘*Ab initio* protein’ database in which all protein sequences were predicted from their corresponding genomic sequences (shown as ‘hmm’ plus number). They are *GpAsh3b*, GpAsh3c, *GpTal1*, *GpSim2*, *GpNPAS3*, *GpId1*, *GpId4*, *GpDec2* and *Hes7*, respectively. There are also 3 GpbHLH protein sequences of which accession numbers were not found in any protein databases. They are *GpKA1*, *GpMist1* and *GpOrphan4*, respectively.

Table 1 showed that, among the 104 bHLH protein sequences deposited in giant panda databases, 58 were annotated in full agreement with our analytical result (shown as the same name in the column of “annotation in GenBank” with that in the column of “gene name”), 33 were annotated differently with our result (shown as a different name in the column of “annotation in GenBank” with that in the column of “gene name”), and 13 were merely predicted proteins (indicated as “hypothetical protein”). Therefore, our work not only newly identified the 13 protein sequences as bHLH family members but also provided additional information for further investigations on the 33 differently annotated bHLH protein sequences.

The coding regions and intron analysis for 107 giant panda bHLH motifs are listed in Table 2. The data of intron analyses showed that there are 47 GpbHLH members with introns in their bHLH motifs. It was found that: (i) 26 GpbHLH members have one intron, among which 13 GpbHLH members have introns in the basic region, 12 have introns in the loop region, and 1 has introns in the helix 2 region. (ii) 19 GpbHLH members have two introns, among which 15 have introns in the basic and loop regions respectively, 3 have introns in the basic and helix 2 regions respectively, and 1 has introns in the helix1 and helix 2 regions respectively. (iii) 2 GpbHLH members have three introns among which two were located in basic region and one was located in helix 2 region. There are altogether 70 introns being identified in the 47 GpbHLH motifs. The longest intron in GpbHLH motif is 45,217 bp (base pairs), and the average length of intron is 4,393 bp. These data are comparable with those of mouse. In mouse, there are also 47 bHLH members having introns in their bHLH motifs. The total number of introns identified is 73, with the longest one of 48,288 bp and the average length of 4,286 bp (data not shown).

**Table 2:** Coding regions, intron location and length of 107 giant panda bHLH motifs.

Family	Gene name	Genomic coding sequence(s)			Intron (location: length)
		Contig No.	Frame	Coding region(s)	
ASCa	*GpAsh1*	NW_003217384.1	+1	937516-937674	
	*GpAsh2*	NW_003217681.1	−2	1024199-1024041	
ASCb	*GpAsh3a*	NW_003217414.1	+1	1397734-1397892	
	*GpAsh3b*	NW_003217343.1	+3	1118238-1118387	
	*GpAsh3c*	NW_003217785.1	−3	1162741-1162583	
MyoD	*GpM*yoD	NW_003218874.1	+3	47166-47321	
	*GpMyoG*	NW_003218226.1	−1	585332-585177	
	*GpMyf5*	NW_003217445.1	−1	705066-704911	
	*GpMyf6*	NW_003217445.1	−3	714220-714065	
E12/E47	*GpTF12*	NW_003217723.1	−3	878591-878430	
	*GpE2A*	NW_003217991.1	−2	930301-930140	
	*GpKA1*	NW_003217991.1	−1	932483-932322	
	*GpTCF4*	NW_003217346.1	+1	1744351-1744512	
Ngn	*GpAth4a*	NW_003218321.1	−3	24207-24049	
	*GpAth4b*	NW_003217318.1	+1	2044831-2044989	
	*GpAth4c*	NW_003217300.1	−3	4043793-4043635	
NeuroD	*GpNDF1*	NW_003217851.1	+3	616848-617006	
	*GpNDF2*	NW_003217447.1	+2	18473-18631	
	*GpAth2*	NW_003217612.1	−1	1092508-1092350	
	*GpAth3*	NW_003219813.1	−3	40317-40159	
Atonal	*GpAth1*	NW_003217374.1	+2	262304-262462	
	*GpAth5*	NW_003217318.1	+3	3168366-3168524	
Mist	*GpMist1*	NW_003218585.1	−1	220417-220259	
Beta3	*GpBeta3b*	NW_003218276.1	+1	208657-208821	
Oligo	*GpOligo1*	NW_003217632.1	−2	1158013-1157843	
	*GpOligo2*	NW_003217632.1	−3	1198932-1198768	
	*GpOligo3*	NW_003217365.1	−3	2603795-2603631	
Net	*GpAth6*	NW_003218843.1	+3	61194-61271	Loop: 8,330 bp
			+2	69602-69682	
Mesp	*GpMesp1*	NW_003217631.1	−3	1622851-1622687	
	*GpPMeso1*	NW_003217360.1	+3	779709-779873	
Twist	*GpTwist*	NW_003217378.1	−2	1057777-1057622	
	*GpDermo1*	NW_003217871.1	−3	1180296-1180141	
Paraxis	*GpParaxis*	NW_003218220.1	−1	310143-310117	Basic: 29,249 bp
			−3	280867-280736	
MyoRa	*GpMyoR*	NW_003217902.1	−2	213023-212865	
	*GpPod1*	NW_003217853.1	+1	879529-879687	
MyoRb	*GpMyoRb1*	NW_003217428.1	−3	2043881-2043723	
	*GpMyoRb2*	NW_003217319.1	−1	759695-759537	
Hand	*GpDHand*	NW_003217296.1	+2	4789691-4789849	
	*GpEHand*	NW_003217471.1	+1	889240-889398	
PTFa	*GpPTFa*	NW_003217305.1	+2	3160973-3161131	
PTFb	*GpPTFb*	NW_003217378.1	−2	1090543-1090385	
SCL	*GpTal1*	NW_003218755.1	+2	402998-403156	
	*GpTal2*	NW_003218606.1	+3	375012-375170	
	*GpLyl1*	NW_003217749.1	−1	440560-440402	
NSCL	*GpHen1*	NW_003218810.1	−1	310980-310822	
	*GpHen2*	NW_003218212.1	+3	118701-118850	No intron (two separate contigs)
		NW_003222115.1	−1	2942-2934	
SRC	*GpSRC1*	NW_003217319.1	−3	2757930-2757913	Basic: 6,972 bp
			−3	2750940-2750785	
	*GpSRC2*	NW_003217553.1	−1	1113592-1113587	Basic: 2,669 bp
			−3	1110917-1110750	
	*GpSRC3*	NW_003218488.1	−3	105991-105983	Basic: 876 bp
			−3	105106-104942	
FIGα	*GpFiga*	NW_003217356.1	+3	2013393-2013428	Basic: 5,738 bp
			+2	2019167-2019289	
Myc	*GpN-Myc*	NW_003217942.1	−3	698202-698044	
	*GpC-Myc*	NW_003217359.1	−3	2278151-2277993	
	*GpL-Myc*	NW_003218583.1	−2	137657-137499	
Mad	*GpMxi1*	NW_003219956.1	−1	80826-80792	Basic: 29,318 bp
			−3	50884-50770	Helix 2: 613 bp
			−1	50156-50148	
	*GpMad1*	NW_003217356.1	−1	2726736-2726732	Basic: 4,015 bp
			−3	2721716-2721687	Basic: 14,277 bp
			−3	2707409-2707295	Helix 2: 1,545 bp
			−3	2705749-2705741	
	*GpMad3*	NW_003218733.1	+2	210165-210168	Basic: 647 bp
			+2	210816-210841	Basic: 128 bp
			+1	210970-211089	Helix 2: 2,020 bp
			+2	213110-213118	
	*GpMad4*	NW_003217437.1	−1	395863-395833	Basic: 563 bp
			−2	390270-390154	Helix 2: 948 bp
			−2	389205-389197	
Mnt	*GpMnt*	NW_003217523.1	+3	590949-590983	Basic: 142 bp
			+1	591126-591237	Helix 2: 5,001 bp
			+1	596239-596247	
Max	*GpMax*	NW_003217331.1	+3	1173984-1174085	Loop: 13,160 bp
			+2	1187246-1187299	
USF	*GpUSF1*	NW_003218870.1	+3	285504-285525	Basic: 121 bp
			+1	285647-285741	Loop: 250 bp
			+3	285990-286040	
	*GpUSF2*	NW_003217744.1	+3	363972-363992	Basic: 6,252 bp
			+1	370243-370338	Loop: 103 bp
			+2	370442-370492	
MITF	*GpMITF*	NW_003218591.1	+3	401976-401997	Basic: 5,362 bp
			+1	407360-407436	Loop: 3,549 bp
			+1	410986-411048	
	*GpTFEb*	NW_003217341.1	+3	3067443-3067464	Basic: 617 bp
			+2	3068082-3068158	Loop: 644 bp
			+1	3068803-3068865	
	*GpTFEc*	NW_003218029.1	+2	296780-296801	Basic: 3,864 bp
			+2	300666-300741	Loop: 4,628 bp
			+1	305370-305433	
	*GpTFE3*	NW_003217513.1	+2	815657-815678	Basic: 172 bp
			+3	815851-815927	Loop: 1,124 bp
			+2	817052-817114	
SREBP	*GpSREBP1*	NW_003217951.1	+1	876259-876357	Loop: 642 bp
			+1	877000-877053	
	*GpSREBP2*	NW_003219081.1	−2	42239-42141	Loop: 1,224 bp
			−2	40916-40863	
AP4	*GpAP4*	NW_003218113.1	+3	891114-891224	Loop: 117 bp
			+3	891342-891386	
MLX	*GpMlx*	NW_003217989.1	−2	869754-869644	Loop: 186 bp
			−2	869457-869404	
	*GpMondoA*	NW_003217304.1	+1	2129887-2129982	Loop: 108 bp
			+1	2130091-2130147	
TF4	*GpTF4*	NW_003217842.1	+2	991667-991717	Helix 1: 260 bp
			+1	991978-992077	Helix 2: 194 bp
			+3	992272-992297	
Clock	*GpClk*	NW_003217618.1	−3	1508321-1508314	Basic: 3,666 bp
			−3	1504647-1504503	
	*GpNPAS2*	NW_003217607.1	−1	787626-787622	Basic: 236 bp
			−3	767385-767238	
ARNT	*GpARNT1*	NW_003217617.1	+1	1009551-1009555	Basic: 2,103 bp
			+3	1011659-1011815	
	*GpARNT2*	NW_003217598.1	−1	1385223-1385219	Basic: 7,467 bp
			−2	1377751-1377595	
Bmal	*GpBmal1*	NW_003218335.1	+2	511835-511839	Basic: 1,225 bp
			+3	513065-513221	
	*GpBmal2*	NW_003217468.1	−2	955835-955831	Basic: 3,125 bp
			−3	952705-952549	
Sim	*GpSim1*	NW_003217828.1	+1	640339-640500	
	*GpSim2*	NW_003217464.1	+2	2035250-2035411	
AHR	*GpAHR1*	NW_003217483.1	+2	1606550-606711	
	*GpAHR2*	NW_003218420.1	−1	471842-471681	
Trh	*GpNPAS3*	NW_003219637.1	−2	62695-2534	
HIF	*GpHif1a*	NW_003217302.1	+2	4505891-4506052	
	*GpHif3a*	NW_003217939.1	−2	1043816-1043655	
	*GpNPAS1*	NW_003217939.1	−2	538490-538344	
	*GpEPAS1*	NW_003217290.1	+2	3566561-3566722	
Emc	*GpId1*	NW_003217538.1	−3	1629899-1629801	
	*GpId2*	NW_003217962.1	+1	246616-246714	
	*GpId3*	NW_003217307.1	−2	256780-256682	
	*GpId4*	NW_003218297.1	+3	402159-402257	
Hey	*GpHerp1*	NW_003218647.1	+3	207195-207212	Basic: 125 bp
			+2	207338-207421	Loop: 276 bp
			+2	207698-207763	
	*GpHerp2*	NW_003217369.1	−1	1395712-1395695	Basic: 132 bp
			−1	1395562-1395479	Loop: 2,267 bp
			−3	1393211-1393446	
	*GpHEYL*	NW_003219577.1	−1	109083-109066	Basic: 1,029 bp
			−1	108036-107953	Loop: 1,266 bp
			−3	106684-106619	
	*GpHey4*	NW_003217325.1	+3	3706089-3706190	Loop: 271 bp
			+1	3706462-3706527	
H/E(spl)	*GpDec1*	NW_003217667.1	+1	608623-608724	Loop: 941 bp
			+3	609666-609731	
	*GpDec2*	NW_003217468.1	+3	2062578-2062679	Loop: 321 bp
			+1	2063002-2063067	
	*GpHes1a*	NW_003219474.1	−2	115790-115785	Basic: 82 bp
			−3	115702-115607	Loop: 96 bp
			−3	115510-115439	
	*GpHes1b*	NW_003218013.1	−1	184110-184105	Basic: 135 bp
			−1	183969-183874	Loop: 194 bp
			−3	183679-183608	
	*GpHes2*	NW_003218027.1	+3	663288-663293	Basic: 86 bp
			+2	663380-663475	Loop: 208 bp
			+3	663684-663755	
	*GpHes3*	NW_003266724.1	−3	806524-806423	Loop: 101 bp
			−2	806321-806253	
	*GpHes7*	NW_003217758.1	+2	1201700-1201801	Loop: 589 bp
			+3	1202391-1202462	
COE	*GpEBF1*	NW_003217293.1	−1	2621749-2621723	Basic: 45,217 bp
			−2	2576505-2576416	Loop: 16,756 bp
			−3	2559659-2559615	
	*GpEBF2*	NW_003217339.1	+3	2341320-2341347	Basic: 19,688 bp
			+2	2361036-2361124	Loop: 1,270 bp
			+3	2362395-2362439	
	*GpEBF3*	NW_003217894.1	+2	583319-583346	Basic: 17,574 bp
			+2	600921-601009	Loop: 5,187 bp
			+2	606197-606241	
Orphan	*GpOrphan2*	NW_003217306.1	−2	2667874-2667746	Helix 2: 1,301 bp
			−1	2666444-2666418	
	*GpOrphan3*	NW_003217988.1	+1	82414-82451	Basic: 16,940 bp
			+3	99392-99509	
	*GpOrphan4*	NW_003218276.1	+2	325310-325347	Basic: 1,985 bp
			+1	327333-327441	

**Note:** Basic, helix 1, loop, and helix 2 regions are delineated as shown in Figure S1.

### The Giant Panda bHLH Repertoire

Compared to the 114 bHLH family members of mouse, it was found that the giant panda has one less member in each of the 7 bHLH families namely Beta3, Mesp, Paraxis, Myc, Hes, COE and Orphan. The missing bHLH family members are Beta3a, Mesp2, Sclerax, S-Myc, Hes5 (or Hes6), EBF4 and Orphan 1, respectively. Based on the available data, it is difficult to say whether giant panda does lack these bHLH genes. At present, there are three mammalian species (human, mouse and rat) of which bHLH family members have been identified and classified [4], [7]. While human has different members with mouse and rat in only 2 bHLH families, i.e. Myc and H/E(spl), it is hard to believe that giant panda could have different members in 7 bHLH families. Moreover, among the 7 family members missing in giant panda, zebrafish and chicken are found to lack only one (S-Myc) and two (S-Myc and EBF4) members, respectively [11], [12]. Therefore, it is thought that additional bHLH members may be found after a new and higher quality version of giant panda genome sequence is released. Nevertheless, given that very little information is available on *bHLH* genes and their functions among bear speices, our data provide a good background information for further studies on regulatory functions of bHLH proteins in giant panda and other bear species.

## Supporting Information

Figure S1 **Alignment of 107 giant panda bHLH family members.** Designation of basic, helix 1, loop and helix 2 follows Ferre-D'Amare *et al.* [25]. The family names and high-order groups have been organized according to Table 1 of Ledent *et al.* [24]. Highly conserved sites are indicated with asterisks on the top. The first five amino acids of NPAS1 were not available due to incompleteness of the correspondent genomic contig sequences.
(TIF)

Figure S2 **Phylogenetic relationship of 107 giant panda and 114 mouse bHLH members.** The tree was constructed with neighbor-joining algorithm with OsRa (the rice bHLH motif sequence of R family) as outgroup. For simplicity, branch lengths of the tree are not proportional to distances between sequences, and bootstrap values less than 50 are not shown. The higher-order group labels are in accordance with Ledent et al. [24].
(TIF)

Figure S3 **In-group phylogenetic analyses of GpAsh1.** (a), (b) and (c) are NJ, MP and ML trees constructed with one giant panda bHLH member (GpAsh1) and nine group A bHLH members from mouse, respectively. In all trees, OsRa was used as the outgroup.
(TIF)

File S1 **Amino acid sequences of 107 giant panda bHLH motifs.** The giant panda bHLH family members are arranged as those in Tables 1 and 2, in which their family assignment, protein and coding region information can be found accordingly.
(DOC)
