# Supplementary figures and images for: A Genome-Wide Survey on Basic Helix-Loop-Helix Transcription Factors in Giant Panda
Source: PLoS One. 2011 Nov 9;6(11):e26878. doi: 10.1371/journal.pone.0026878 (PMC3212526; doi:10.1371/journal.pone.0026878)

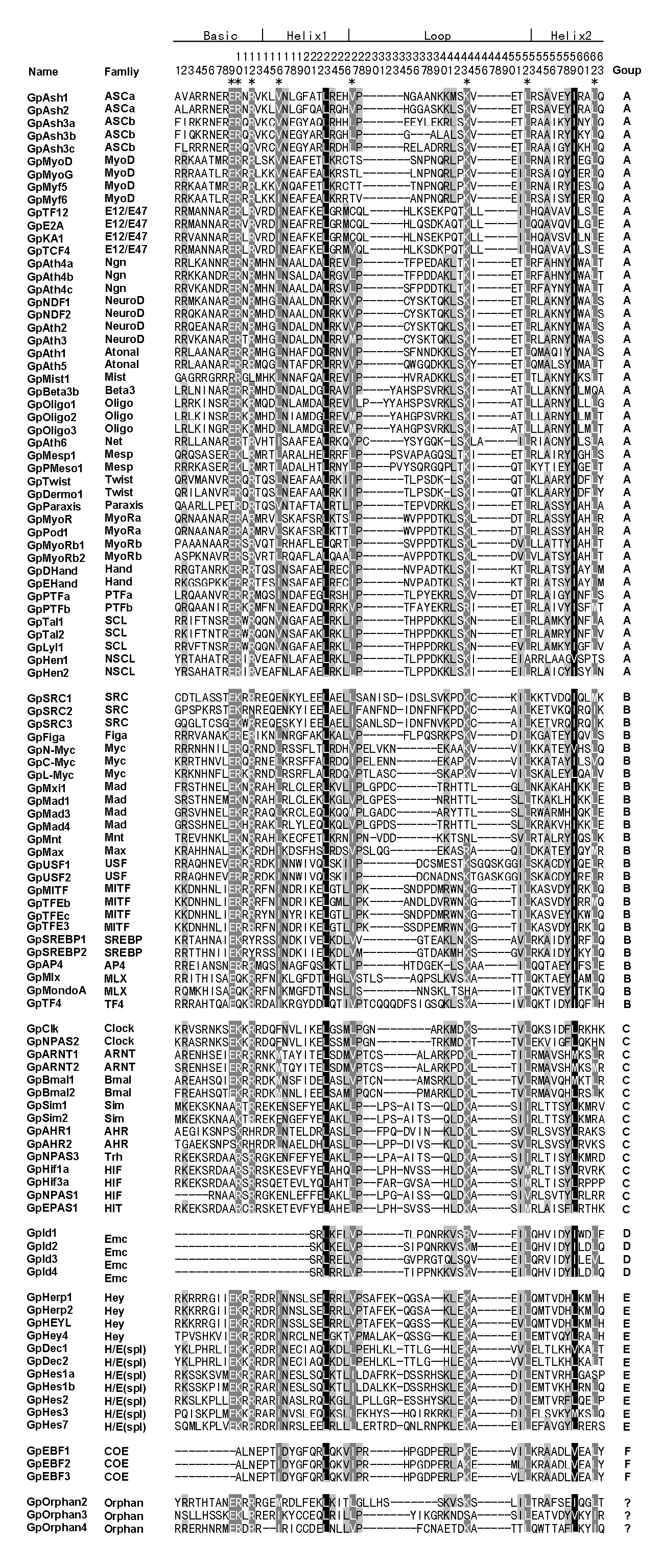

Supplement: Figure S1 — Alignment of 107 giant panda bHLH family members. Designation of basic, helix 1, loop and helix 2 follows Ferre-D'Amare et al. [25]. The family names and high-order groups have been organized according to Table 1 of Ledent et al. [24]. Highly conserved sites are indicated with asterisks on the top. The first five amino acids of NPAS1 were not available due to incompleteness of the correspondent genomic contig sequences. (TIF) [file pone.0026878.s001.tif]

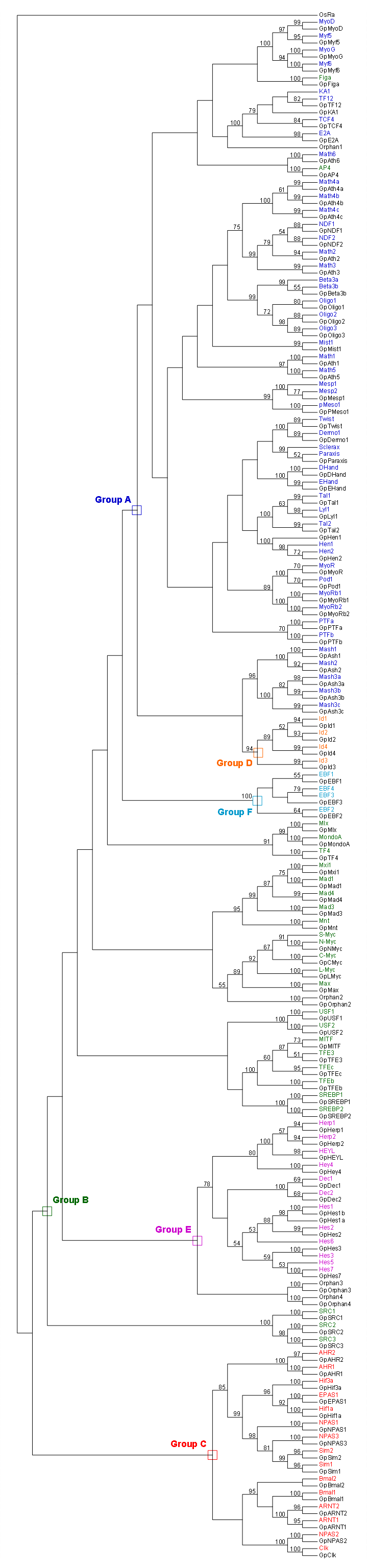

Supplement: Figure S2 — Phylogenetic relationship of 107 giant panda and 114 mouse bHLH members. The tree was constructed with neighbor-joining algorithm with OsRa (the rice bHLH motif sequence of R family) as outgroup. For simplicity, branch lengths of the tree are not proportional to distances between sequences, and bootstrap values less than 50 are not shown. The higher-order group labels are in accordance with Ledent et al. [24]. (TIF) [file pone.0026878.s002.tif]

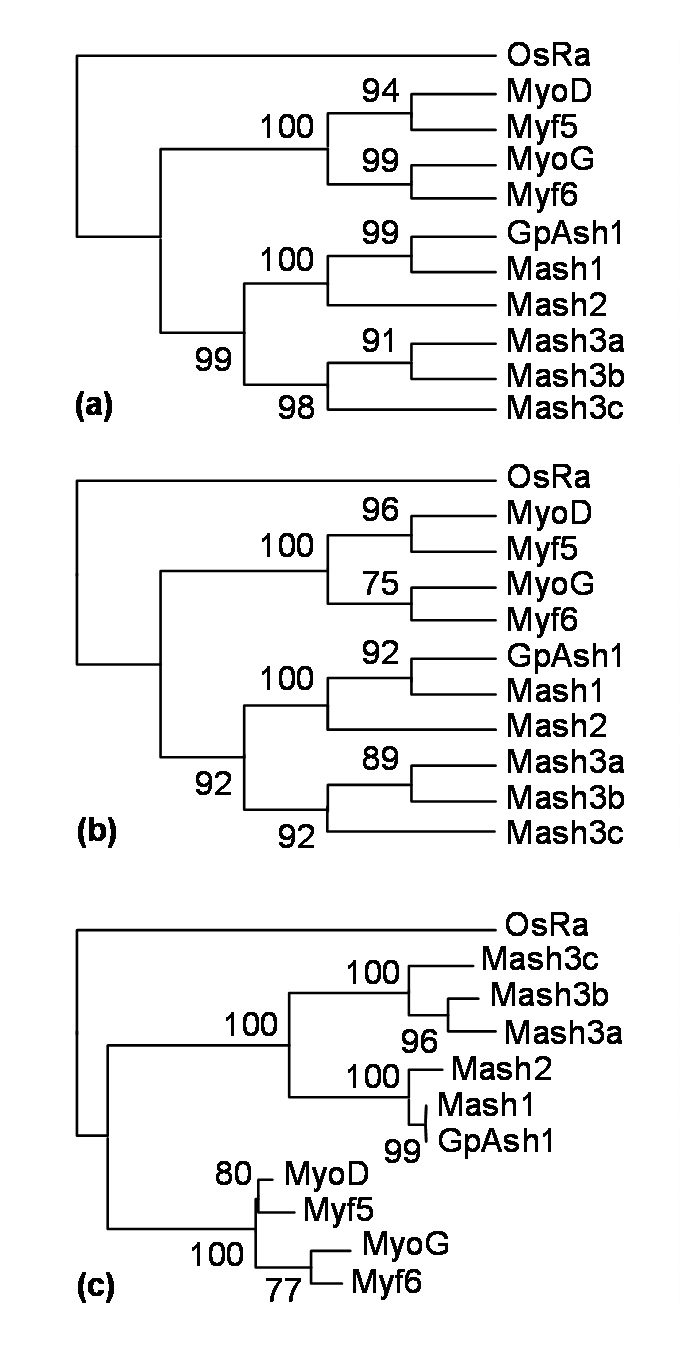

Supplement: Figure S3 — In-group phylogenetic analyses of GpAsh1. (a), (b) and (c) are NJ, MP and ML trees constructed with one giant panda bHLH member (GpAsh1) and nine group A bHLH members from mouse, respectively. In all trees, OsRa was used as the outgroup. (TIF) [file pone.0026878.s003.tif]
