# Supplementary material for: A Genome-Wide Survey on Basic Helix-Loop-Helix Transcription Factors in Giant Panda
Source: PLoS One. 2011 Nov 9;6(11):e26878. doi: 10.1371/journal.pone.0026878 (PMC3212526; doi:10.1371/journal.pone.0026878)
Supplement: File S1 — Amino acid sequences of 107 giant panda bHLH motifs. The giant panda bHLH family members are arranged as those in Tables 1 and 2, in which their family assignment, protein and coding region information can be found accordingly. (DOC) [file pone.0026878.s004.doc]

>GpAsh1

AVARRNERERNRVKLVNLGFATLREHVPNGAANKKMSKVETLRSAVEYIRALQ

>GpAsh2

ALARRNERERNRVKLVNLGFQALRQHVPHGGASKKLSKVETLRSAVEYIRALQ

>GpAsh3a

FIRKRNERERQRVKCVNEGYAQLRHHLPEEYLEKRLSKVETLRAAIKYINYLQ

>GpAsh3b

FIQKRNERERQRVKCVNEGYARLRGHLPGALALSKVETLRAAIRYIKYLQ

>GpAsh3c

FLRRRNERERQRVRCVNEGYARLRDHLPRELADRRLSKVETLRAAIGYIKHLQ

>GpMyoD

RRKAATMRERRRLSKVNEAFETLKRCTSSNPNQRLPKVEILRNAIRYIEGLQ

>GpMyoG

RRRAATLREKRRLKKVNEAFEALKRSTLLNPNQRLPKVEILRSAIQYIERLQ

>GpMyf5

RRKAATMRERRRLKKVNQAFETLKRCTTTNPNQRLPKVEILRNAIRYIESLQ

>GpMyf6

RRKAATLRERRRLKKINEAFEALKRRTVANPNQRLPKVEILRSAISYIERLQ

>GpTF12

RRMANNARERLRVRDINEAFKELGRMCQLHLKSEKPQTKLLILHQAVAVILSLE

>GpE2A

RRMANNARERVRVRDINEAFRELGRMCQLHLQSDKAQTKLLILQQAVQVILGLE

>GpKA1

RRVANNARERLRVRDINEAFKELGRMCQLHLNSEKPQTKLLILHQAVSVILNLE

>GpTCF4

RRMANNARERLRVRDINEAFKELGRMVQHLKSDKPQTKLLILHQAVAVILSLE

>GpAth4a

RRLKANNRERNRMHNLNAALDALREVLPTFPEDAKLTKIETLRFAHNYIWALT

>GpAth4b

RRKKANDRERNRMHNLNSALDALRGVLPTFPDDAKLTKIETLRFAHNYIWALT

>GpAth4c

RRVKANDRERNRMHNLNAALDALRSVLPSFPDDTKLTKIETLRFAYNYIWALT

>GpNDF1

RRMKANARERNRMHGLNAALDNLRKVVPCYSKTQKLSKIETLRLAKNYIWALS

>GpNDF2

RRQKANARERNRMHDLNAALDNLRKVVPCYSKTQKLSKIETLRLAKNYIWALS

>GpAth2

RRQEANARERNRMHGLNDALDNLRKVVPCYSKTQKLSKIETLRLAKNYIWALS

>GpAth3

RRVKANARERTRMHGLNDALDNLRRVMPCYSKTQKLSKIETLRLARNYIWALS

>GpAth1

RRLAANARERRRMHGLNHAFDQLRNVIPSFNNDKKLSKYETLQMAQIYINALS

>GpAth5

RRLAANARERRRMQGLNTAFDRLRRVVPQWGQDKKLSKYETLQMALSYIMALT

>GpMist1

GAGRRGRRRRGLMHKLNNAFQALREVIPHVRADKKLSKIETLTLAKNYIKSLT

>GpBeta3b

LRLNINARERRRMHDLNDALDGLRAVIPYAHSPSVRKLSKIATLLLAKNYILMQA

>GpOligo1

LRRKINSRERKRMQDLNLAMDALREVILPYYAHSPSVRKLSKIATLLLARNYILLLG

>GpOligo2

LRLKINSRERKRMHDLNIAMDGLREVMPYAHGPSVRKLSKIATLLLARNYILMLT

>GpOligo3

LRLKINGRERKRMHDLNLAMDGLREVMPYAHGPSVRKLSKIATLLLARNYILMLT

>GpAth6

RRLLANARERTRVHTISAAFEALRKQVPCYSYGQK-LSKL-ILRIACNYILSLA

>GpMesp1

QRQSASEREKLRMRTLARALHELRRFLPPSVAPAGQSLTKIETLRLAIRYIGHLS

>GpPMeso1

RRRKASEREKLRMRTLADALHTLRNYLPPVYSQRGQPLTKIQTLKYTIEYIGELT

>GpTwist

QRVMANVRERQRTQSLNEAFAALRKIIPTLPSDK-LSKIQTLKLAARYIDFLY

>GpDermo1

QRILANVRERQRTQSLNEAFAALRKIIPTLPSDK-LSKIQTLKLAARYIDFLY

>GpParaxis

QAARLLPETRDRTQSVNTAFTALRTLIPTEPVDRKLSKIETLRLASSYIAHLA

>GpMyoR

QRNAANARERARMRVLSKAFSRLKTSLPWVPPDTKLSKLDTLRLASSYIAHLR

>GpPod1

QRNAANARERARMRVLSKAFSRLKTTLPWVPPDTKLSKLDTLRLASSYIAHLR

>GpMyoRb1

PAAANAARERSRVQTLRHAFLELQRTLPSVPPDTKLSKLDVLLLATTYIAHLT

>GpMyoRb2

ASPKNAVRERSRVRTLRQAFLALQAALPAVPPDTKLSKLDVLVLATSYIAHLT

>GpDHand

RRGTANRKERRRTQSINSAFAELRECIPNVPADTKLSKIKTLRLATSYIAYLM

>GpEHand

RKGSGPKKERRRTESINSAFAELRECIPNVPADTKLSKIKTLRLATSYIAYLM

>GpPTFa

LRQAANVRERRRMQSINDAFEGLRSHIPTLPYEKRLSKVDTLRLAIGYINFLS

>GpPTFb

QRQAANIRERKRMFNLNEAFDQLRRKVPTFAYEKRLSRIETLRLAIVYISFMT

>GpTal1

RRIFTNSRERWRQQNVNGAFAELRKLIPTHPPDKKLSKNEILRLAMKYINFLA

>GpTal2

RKIFTNTRERWRQQNVNSAFAKLRKLIPTHPPDKKLSKNETLRLAMRYINFLV

>GpLyl1

RRVFTNSRERWRQQNVNGAFAELRKLLPTHPPDRKLSKNEVLRLAMKYIGFLV

>GpHen1

YRTAHATRERIRVEAFNLAFAELRKLLPTLPPDKKLSKIEIARRLAAGVSPTS

>GpHen2

YRSAHATRERIRVEAFNLAFAELRKLLPTLPPDKKLSKIEILRLAICYISYLN

>GpSRC1

CDTLASSTEKRRREQENKYLEELAELLSANISDIDSLSVKPDKCKILKKTVDQIQLMK

>GpSRC2

GPSPKRSTEKRNREQENKYIEELAELIFANFNDIDNFNFKPDKCAILKETVKQIRQIK

>GpSRC3

GQGLTCSGEKWRREQESKYIEELAELISANLSDIDNFNVKPDKCAILKETVRQIRQIK

>GpFiga

RRRVANAKERERIKNLNRGFAKLKALVPFLPQSRKPSKVDILKGATEYIQVLS

>GpN-Myc

RRRNHNILERQRRNDLRSSFLTLRDHVPELVKNEKAAKVVILKKATEYVHSLQ

>GpC-Myc

KRRTHNVLERQRRNELKRSFFALRDQIPELENNEKAPKVVILKKATAYILSVQ

>GpL-Myc

KRKNHNFLERKRRNDLRSRFLALRDQVPTLASCSKAPKVVILSKALEYLQALV

>GpMxi1

FRSTHNELEKNRRAHLRLCLERLKVLIPLGPDCTRHTTLGLLNKAKAHIKKLE

>GpMad1

SRSTHNEMEKNRRAHLRLCLEKLKGLVPLGPESNRHTTLSLLTKAKLHIKKLE

>GpMad3

GRSVHNELEKRRRAQLKRCLEQLKQQMPLGADCARYTTLSLLRWARMHIQKLE

>GpMad4

GRSSHNELEKHRRAKLRLYLEQLKQLVPLGPDSTRHTTLSLLKRAKVHIKKLE

>GpMnt

TREVHNKLEKNRRAHLKECFETLKRNIPN-VDDKKTSNLSVLRTALRYIQSLK

>GpMax

KRAHHNALERKRRDHIKDSFHSLRDSVPSLQGEKASRAQILDKATEYIQYMR

>GpUSF1

RRAQHNEVERRRRDKINNWIVQLSKIIPDCSMESTKSGQSKGGILSKACDYIQELR

>GpUSF2

RRAQHNEVERRRRDKINNWIVQLSKIIPDCNADNSKTGASKGGILSKACDYIRELR

>GpMITF

KKDNHNLIERRRRFNINDRIKELGTLIPKSNDPDMRWNKGTILKASVDYIRKLQ

>GpTFEb

KKDNHNLIERRRRFNINDRIKELGMLIPKANDLDVRWNKGTILKASVDYIRRMQ

>GpTFEc

KKDNHNLIERRRRYNINYRIKELGTLIPKSNDPDMRWNKGTILKASVEYIKWLQ

>GpTFE3

KKDNHNLIERRRRFNINDRIKELGTLIPKSSDPEMRWNKGTILKASVDYIRKLQ

>GpSREBP1

KRTAHNAIEKRYRSSINDKIVELKDLVVGTEAKLNKSAVLRKAIDYIRFLQ

>GpSREBP2

RRTTHNIIEKRYRSSINDKIIELKDLVMGTDAKMHKSGVLRKAIDYIKYLQ

>GpAP4

RREIANSNERRRMQSINAGFQSLKTLIPHTDGEKLSKAAILQQTAEYIFSLE

>GpMlx

RRITHISAEQKRRFNIKLGFDTLHGLVSTLSAQPSLKVSKATTLQKTAEYIAMLQ

>GpMondoA

RQMKHISAEQKRRFNIKMGFDTLNSLISNNSKLTSHAITLQKTVEYITKLQ

>GpTF4

RRRAHTQAEQKRRDAIKRGYDDLQTIVPTCQQQDFSIGSQKLSKAIVLQKTIDYIQFLH

>GpClk

KRVSRNKSEKKRRDQFNVLIKELGSMLPGNARKMDKSTVLQKSIDFLRKHK

>GpNPAS2

KRASRNKSEKKRRDQFNVLIKELSSMLPGNTRKMDKTTVLEKVIGFLQKHN

>GpARNT1

ARENHSEIERRRRNKMTAYITELSDMVPTCSALARKPDKLTILRMAVSHMKSLR

>GpARNT2

SRENHSEIERRRRNKMTQYITELSDMVPTCSALARKPDKLTILRMAVSHMKSMR

>GpBmal1

AREAHSQIEKRRRDKMNSFIDELASLVPTCNAMSRKLDKLTVLRMAVQHMKTLR

>GpBmal2

FREAHSQTEKRRRDKMNNLIEELSAMIPQCNPMARKLDKLTVLRMAVQHLRSLK

>GpSim1

MKEKSKNAARTRREKENSEFYELAKLLPLPSAITSQLDKASIIRLTTSYLKMRV

>GpSim2

MKEKSKNAAKTRREKENGEFYELAKLLPLPSAITSQLDKASIIRLTTSYLKMRA

>GpAHR1

AEGIKSNPSKRHRDRLNTELDRLASLLPFPQDVINKLDKLSVLRLSVSYLRAKS

>GpAHR2

TGAEKSNPSKRHRDRLNAELDHLASLLPLPPDIVSKLDKLSVLRLSVSYLRVKS

>GpNPAS3

RKEKSRDAARSRRGKENFEFYELAKLLPLPAAITSQLDKASIIRLTISYLKMRD

>GpHif1a

RKEKSRDAARSRRSKESEVFYELAHQLPLPHNVSSHLDKASVMRLTISYLRVRK

>GpHif3a

RKEKSRDAARSRRSQETEVLYQLAHTLPFARGVSAHLDKASIMRLTISYLRPPP

>GpNPAS1

RNAARSRRGKENLEFFELAKLLPLPGAISSQLDKASIVRLSVTYLRLRR

>Gp EPAS1

RKEKSRDAARCRRSKETEVFYELAHELPLPHSVSSHLDKASIMRLAISFLRTHK

>GpId1

SRLKELVPTLPQNRKVSRVEILQHVIDYIWDLE

>GpId2

SKLKELVPSIPQNRKVSKMEILQHVIDYILDLQ

>GpId3

SRLRELVPGVPRGTQLSQVEILQRVIDYILEVL

>GpId4

SRLRRLVPTIPPNKKVSKVEILQHVIDYILDLQ

>GpHerp1

RKRRRGIIEKRRRDRINNSLSELRRLVPSAFEKQGSAKLEKAEILQMTVDHLKMLH

>GpHerp2

RKKRRGIIEKRRRDRINNSLSELRRLVPTAFEKQGSAKLEKAEILQMTVDHLKMLQ

>GpHEYL

RKKRRGIIEKRRRDRINSSLSELRRLVPTAFEKQGSSKLEKAEVLQMTVDHLKMLH

>GpHey4

TPVSHKVIEKRRRDRINRCLNELGKTVPMALAKQSSGKLEKAEILEMTVQYLRALH

>GpDec1

YKLPHRLIEKKRRDRINECIAQLKDLLPEHLKLTTLGHLEKAVVLELTLKHVKALT

>GpDec2

YKLPHRLIEKKRRDRINECIAQLKDLLPEHLKLTTLGHLEKAVVLELTLKHLKALT

>GpHes1a

RKSSKSVMEKRRRARINESLSQLKTLILDAFRKDSSRHSKLEKADILENTVRHLGASP

>GpHes1b

RKSSKPIMEKRRRARINESLSQLKTLILDALKKDSSRHSKLEKADILEMTVKHLRNLQ

>GpHes2

RKSLKPLLEKRRRARINASLRQLKGLILPLLGRESSHYSKLEKADILEMTVRFLQELP

>GpHes3

PQISKPLMEKKRRARINVSLEQLKSLLEKHYSHQIRKRKLEKADILELSVKYMKSLQ

>GpHes7

SQMLKPLVEKRRRDRINRSLEELRLLLLERTRDQNLRNPKLEKAEILEFAVGYLRERS

>GpEBF1

ALNEPTIDYGFQRLQKVIPRHPGDPERLPKEVILKRAADLVEALY

>GpEBF2

ALNEPTIDYGFQRLQKVIPRHPGDPERLAKEMLLKRAADLVEALY

>GpEBF3

ALNEPTIDYGFQRLQKVIPRHPGDPERLPKEVLLKRAADLVEALY

>GpOrphan2

YRRTHTANERRRRGEMRDLFEKLKITLGLLHSSKVSKSLILTRAFSEIQGLT

>GpOrphan3

NSLLHSSKEKLRRERIKYCCEQLRILLPYIKGRKNDSASILEATVDYVKYIR

>GpOrphan4

RRERHNRMERDRRIRICCDELNLLVPFCNAETDKATTLQWTTAFLKYIQ
